# MutT homologue 1 (MTH1) removes N6-methyl-dATP from the dNTP pool

**DOI:** 10.1074/jbc.RA120.012636

**Published:** 2020-03-06

**Authors:** Emma Rose Scaletti, Karl S. Vallin, Lars Bräutigam, Antonio Sarno, Ulrika Warpman Berglund, Thomas Helleday, Pål Stenmark, Ann-Sofie Jemth

**Affiliations:** ‡ Department of Biochemistry and Biophysics, Stockholm University S-106 91, Stockholm, Sweden; ¶ Science for Life Laboratory, Department of Oncology-Pathology, Karolinska Institutet, S-171 76 Stockholm, Sweden; ‖ Department of Clinical and Molecular Medicine, Norwegian University of Science and Technology, 7491 Trondheim, Norway; ** Department of Pathology, St. Olavs Hospital, 7006 Trondheim, Norway; ‡‡ Weston Park Cancer Centre, Department of Oncology and Metabolism, University of Sheffield, Sheffield S10 2RX, United Kingdom; § Department of Experimental Medical Science, Lund University, Lund 221 00, Sweden

**Keywords:** crystal structure, X-ray crystallography, enzyme catalysis, substrate specificity, enzyme kinetics, nucleoside/nucleotide metabolism, hydrolase, epigenetics, methylation, MutT homologue 1 (MTH1), N6-methyl-dATP, nucleotide hydrolysis, Nudix hydrolase 1 (NUDT1)

## Abstract

MutT homologue 1 (MTH1) removes oxidized nucleotides from the nucleotide pool and thereby prevents their incorporation into the genome and thereby reduces genotoxicity. We previously reported that MTH1 is an efficient catalyst of O6-methyl-dGTP hydrolysis suggesting that MTH1 may also sanitize the nucleotide pool from other methylated nucleotides. We here show that MTH1 efficiently catalyzes the hydrolysis of N6-methyl-dATP to N6-methyl-dAMP and further report that N6-methylation of dATP drastically increases the MTH1 activity. We also observed MTH1 activity with N6-methyl-ATP, albeit at a lower level. We show that N6-methyl-dATP is incorporated into DNA *in vivo*, as indicated by increased N6-methyl-dA DNA levels in embryos developed from MTH1 knock-out zebrafish eggs microinjected with N6-methyl-dATP compared with noninjected embryos. N6-methyl-dATP activity is present in MTH1 homologues from distantly related vertebrates, suggesting evolutionary conservation and indicating that this activity is important. Of note, N6-methyl-dATP activity is unique to MTH1 among related NUDIX hydrolases. Moreover, we present the structure of N6-methyl-dAMP–bound human MTH1, revealing that the N6-methyl group is accommodated within a hydrophobic active-site subpocket explaining why N6-methyl-dATP is a good MTH1 substrate. N6-methylation of DNA and RNA has been reported to have epigenetic roles and to affect mRNA metabolism. We propose that MTH1 acts in concert with adenosine deaminase-like protein isoform 1 (ADAL1) to prevent incorporation of N6-methyl-(d)ATP into DNA and RNA. This would hinder potential dysregulation of epigenetic control and RNA metabolism via conversion of N6-methyl-(d)ATP to N6-methyl-(d)AMP, followed by ADAL1-catalyzed deamination producing (d)IMP that can enter the nucleotide salvage pathway.

## Introduction

Human MutT homologue 1 (MTH1)^3^ belongs to the NUDIX family of proteins (1) and catalyzes the hydrolysis of the oxidized purine nucleoside triphosphates 8-oxo-dGTP, 8-oxo-GTP, 2-OH-dATP, and 2-OH-ATP into their corresponding monophosphates (2). Removal of oxidized purine nucleoside triphosphates from the free nucleotide pool through MTH1-catalyzed hydrolysis prevents misincorporation of these nucleotides into DNA and RNA and reduces genotoxicity (3). Cells from MTH1 knockout (MTH1KO) mice display a 2-fold increased mutation rate. However, these mice grow old and a higher tumor burden compared with MTH1 wildtype (MTH1WT) mice is only observed in old MTH1KO mice, suggesting that MTH1 is dispensible in the short term under normal conditions (4). Mice that overexpress MTH1 live longer, have a better memory, are more social at an old age, and have lower levels of 8-oxo-dG in their DNA compared with both MTH1WT and MTH1KO mice, supporting a correlation between 8-oxo-dG levels in DNA and aging (5). MTH1 is required for cancer cell survival, yet seems to be dispensible for growth of untransformed cells (6, 7). This is thought to be due to the elevated reactive oxygen species load present in cancer cells compared with normal cells, which damages free nucleotides. MTH1 was proposed to be a good target for specifically targeting cancer cells and several potent small molecule MTH1 inhibitors were developed (6, 8). Interestingly, not all developed inhibitors of MTH1 have the same cellular effect and a growing body of evidence suggests that MTH1 has other cellular roles in addition to sanitizing the oxidized nucleotide pool (7, 9). We recently reported that human MTH1, in addition to hydrolyzing oxidized purine nucleoside triphosphates, also is a highly efficient catalyst of O6-methyl-dGTP hydrolysis (10). This prompted us to test other methylated nucleotides as MTH1 substrates and we here present N6-methyl-dATP and N6-methyl-ATP as novel substrates for MTH1. We show that human MTH1 effectively catalyzes the hydrolysis of N6-methyl-dATP to N6-methyl-dAMP and also catalyzes N6-methyl-ATP hydrolysis, albeit at a lower rate. This N6-methyl-dATP hydrolysis activity was present in all the animal MTH1 homologues we tested, ranging from zebrafish to humans. Furthermore, we demonstrate that N6-methyl-dATP present in the nucleotide pool is incorporated into DNA *in vivo* in an MTH1-dependent manner. Because N6-methylation of adenine has been described to affect gene expression in eukaryotic cells (11), as well as to influence RNA processes such as transcription, splicing, translation, and stability (12), we speculate that this novel MTH1 activity may help to safeguard against dysregulation of the cellular epigenome and epitranscriptome. This study expands the substrate collection of human MTH1 and emphasizes the importance of MTH1 in sanitizing the nucleotide pool by catalyzing the hydrolysis of both methylated and oxidized nucleotides.

## Results

### MTH1 catalyzes the hydrolysis of N6-methyl-dATP to N6-methyl-dAMP

**Figure 1.**
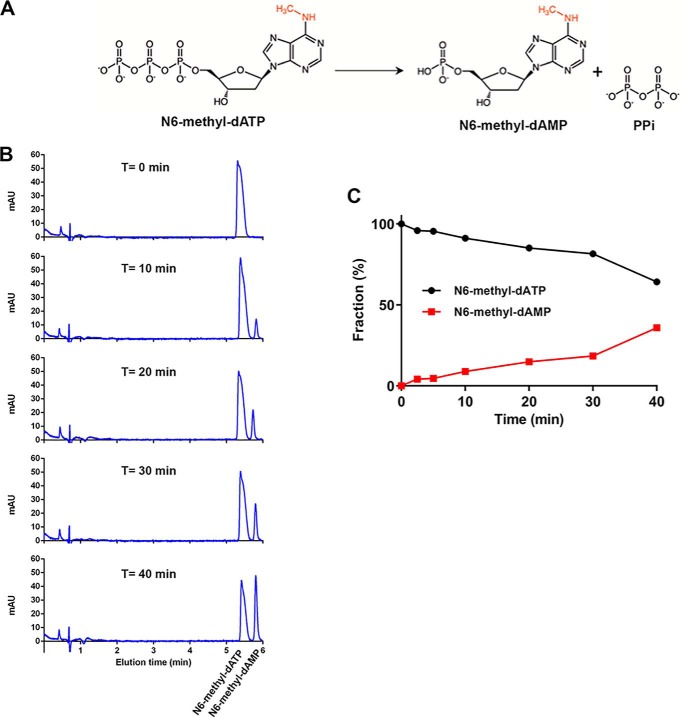
**MTH1 catalyzes the hydrolysis of N6-methyl-dATP.** *A,* MTH1 catalyzes the hydrolysis of N6-methyl-dATP to N6-methyl-dAMP and PP_i_. *B,* time course hydrolysis of N6-methyl-dATP (1 mm) catalyzed by MTH1 (20 nm) was monitored by separation of reaction samples incubated 0–40 min at 22 °C on a Hypercarb column using HPLC coupled to MS. Reaction substrate and product was detected at 254 nm and the mass of the product N6-methyl-dAMP was clearly observed by mass detection. *C,* graph showing the fraction of N6-methyl-dATP and N6-methyl-dAMP in percent after various times of hydrolysis based on the respective area under the curve of the peaks in the corresponding HPLC chromatogram.

**Figure 2.**
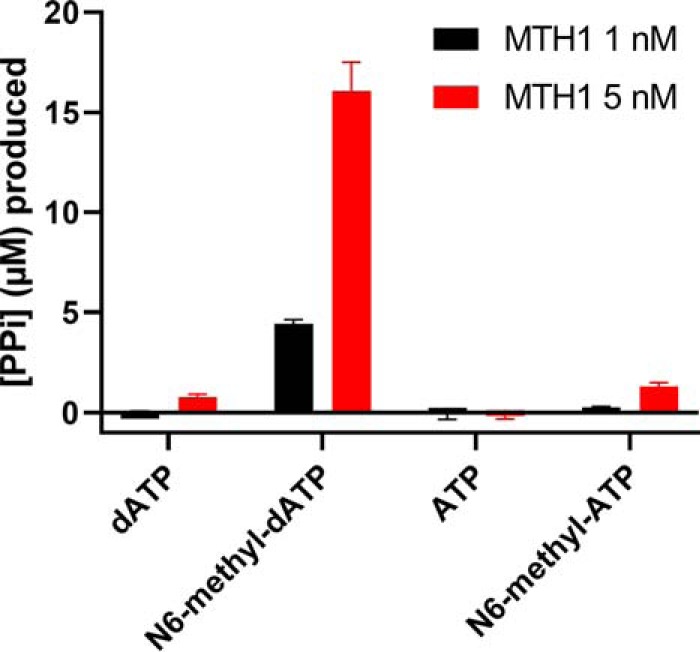
**MTH1 activity with N6-methyl-dATP and N6-methyl-ATP compared with dATP.** Activity of 1 and 5 nm MTH1 was tested with 50 μm N6-methyl-dATP, dATP, ATP, and N6-methyl-ATP in MTH1 reaction buffer (pH 8.0) at 22 °C. Reaction time was 30 min and 0.2 units/ml of PPase was used to generate P_i_ from produced PP_i_. P_i_ was detected by addition of malachite green reagent followed by measurement of the absorbance at 630 nm. Controls with PPase only was included and background signal was subtracted from the assay data. A P_i_ standard curve was included on the plate enabling determination of the concentration of formed PP_i_. Graph shows mean ± S.D. from one experiment performed in quadruplicate.

The results of HPLC and MS analysis of reaction products after MTH1 incubation with N6-methyl-dATP over differing lengths of time, show that MTH1 catalyzes the hydrolysis of N6-methyl-dATP to N6-methyl-dAMP and inorganic pyrophosphate (PP_i_) (Fig. 1*A*). The amount of formed N6-methyl-dAMP increases in a time-dependent manner (Fig. 1, *B* and *C*), with the loss of PP_i_ clearly observed by detection of the mass of N6-methyl-dAMP. N6-methyl-dATP was found to be stable over time under the reaction conditions used, with no detected hydrolysis observed in the absence of enzyme for up to at least 6 h. To further evaluate the activity of MTH1 with N6-methyl-dATP we determined the specific activities of MTH1 with 50 μm dATP, N6-methyl-dATP, ATP, and N6-methyl-ATP. We found the specific activity with N6-methyl-dATP to be ∼20-fold higher than the activity with nonmethylated dATP (Fig. 2). MTH1 also displays a low but measurable activity with N6-methyl-ATP, whereas no detectable activity with ATP was observed. The specific activity with N6-methyl-ATP is ∼11 times lower than the activity with N6-methyl-dATP showing that MTH1 clearly prefers deoxyribonucleotides over ribonucleotides as substrates. Hydrolysis of the selected nucleotides was tested at two different MTH1 concentrations, showing a clear dependence of substrate hydrolysis on MTH1 concentration

### MTH1 is an efficient catalyst of N6-methyl-dATP hydrolysis

**Figure 3.**
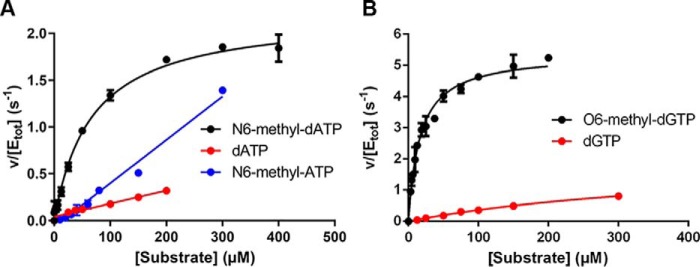
**Kinetic characterization of MTH1-catalyzed hydrolysis of dATP, N6-methyl-dATP, N6-methyl-ATP, dGTP, and O6-methyl-dGTP.** *A,* substrate saturation curves of MTH1 (1 nm) were produced using MTH1 reaction buffer (pH 7.5). Initial rates were determined at dATP concentrations varied between 0 and 200 μm and between 0 and 300 μm for N6-methyl-dATP and N6-methyl-ATP, respectively, and for dGTP and O6-methyl-dGTP (*B*) using 0–300 and 0–200 μm, respectively. Formed PP_i_ was detected using PPiLight^TM^ Inorganic Pyrophosphate Assay (Lonza) and the assay signal was converted to concentration of PP_i_ by including a PP_i_ standard curve on the assay plate.

Substrate saturation curves of MTH1 were generated for dATP, N6-methyl-dATP, N6-methyl-ATP, dGTP, and O6-methyl-dGTP (Fig. 3, *A* and *B*) at close to physiological pH (pH 7.5), and kinetic parameters were determined (Table 1). Addition of the N6-methyl group to dATP increases the catalytic efficiency (*k*_cat_/*K_m_*) ∼17-fold. Similarly, methylation of dGTP at the O6 position increases the *k*_cat_/*K_m_* value considerably. It is difficult to estimate the effect of N6-methylation on *k*_cat_ and *K_m_* values because these values cannot be determined for dATP due to no substrate saturation in the concentration range used. However, it is likely that the *K_m_* value of MTH1 for dATP is higher than 200 μm, which was the highest dATP concentration used and which was still located in the linear part of the saturation curve. Consequently, the *K_m_* value for dATP is likely to be at least 1 order of magnitude higher than the *K_m_* value for N6-methyl-dATP. The *k*_cat_ value of MTH1 with N6-methyl-dATP (2.0 s^−1^) is 2.7-fold lower compared with the k_cat_ value of MTH1 with O6-methyl-dGTP (5.4 s^−1^) but 2-fold higher than the *k*_cat_ value for dGTP (1.0 s^−1^). The *K_m_* value of MTH1 is ∼2.5 times lower for O6-methyl-dGTP compared with that for N6-methyl-dATP. This results in a *k*_cat_/*K_m_* value for O6-methyl-dGTP that is ∼6-fold higher compared with N6-methyl-dATP. This may partly be attributable to the lower *K_m_* value of MTH1 for dGTP compared with dATP, which may reflect a higher affinity for guanine compared with adenine. No substrate saturation was obtained with N6-methyl-ATP in the concentration range used, meaning the determination of *k*_cat_ and *K_m_* was not possible. However, the *k*_cat_/*K_m_* value was determined from the slope of the linear part of the saturation curve to be 4100 m^−1^ s^−1^. This value is more than 1 order of magnitude lower than the corresponding value for N6-methyl-dATP, strongly supporting a preference of MTH1 for deoxyribonucleotides over ribonucleotides. Altogether, the kinetic analysis demonstrates that methylation of both N6 of dATP and O6 of dGTP greatly improves them as MTH1 substrates and that this enzyme catalyzes the hydrolysis of these methylated substrates with high catalytic efficiency. This result shows that the active site of MTH1 has the capability to accommodate N6-methylated dATP and O6-methylated dGTP in addition to the previously described oxidatively modified adenosine and guanine nucleoside triphosphates (13, 14).

**Table 1:** **Kinetic parameters of MTH1 for canonical and methylated substrates** Kinetic parameters were determined by fitting the Michaelis-Menten equation to determined initial rates using GraphPad Prism. Average and S.D. of determined kinetic parameters from several independent experiments (*n*) with initial rates in at least duplicates are presented.

Substrate	*k*_cat_	*K_m_*	*k*_cat_/*K_m_*	*n*
	*s*^−*1*^	μ*m*	*m*^−*1*^ *s*^−*1*^	
N6-methyl-dATP	2.0 ± 0.4	40.9 ± 8.2	50,300 ± 15,700	4
dATP	-	-	3,000 ± 200	3
N6-methyl-ATP	-	-	4,100 ± 800	2
dGTP	1.0 ± 0.4	154 ± 92	7,100 ± 1,900	3
O6-methyl-dGTP	5.4 ± 0.4	16.5 ± 0.3	320,100 ± 26,100	3

### N6-methyl-dATP activity is unique to MTH1 among closely related NUDIX enzymes

**Figure 4.**
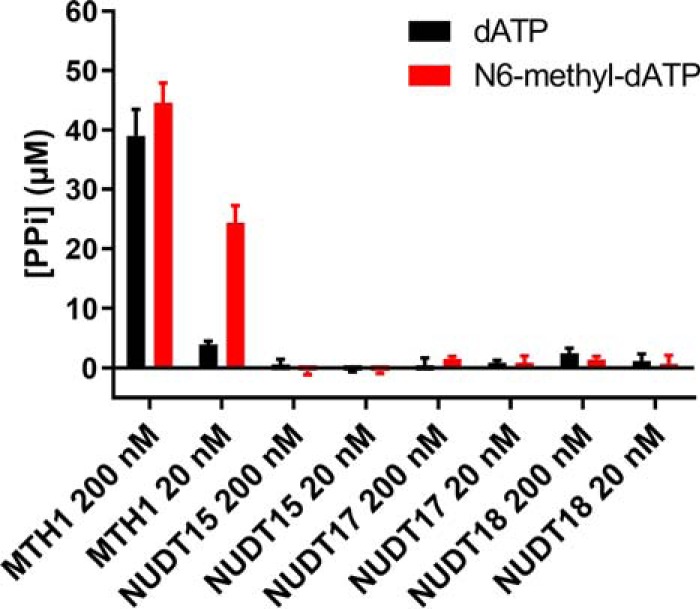
**Activity with N6-methyl-dATP is unique to MTH1 within human NUDIX subfamily.** Activities of human NUDIX enzyme (MTH1, NUDT15, NUDT17, and NUDT18) were assayed with data points in quadruplicate with 50 μm dATP or N6-methyl-dATP at 20 and 200 nm enzyme in MTH1 reaction buffer (pH 8.0). 0.2 units/ml of PPase was used to convert formed PP_i_ to P_i_ that was detected using malachite green reagent and measurement of absorbance at 630 nm. Graph shows mean ± S.D. from one experiment performed in triplicate.

NUDT15, NUDT17, NUDT18, NUDT5, and NUDT14 group together with MTH1 in the phylogenetic tree of NUDIX proteins (15–17). MTH1, NUDT15, and NUDT18 cluster together when performing a hierarchical clustering of substrate activities of human NUDIX enzymes (17). Because these enzymes may share substrates, the activities of MTH1, NUDT15, NUDT17, and NUDT18 with N6-methyl-dATP and dATP, were tested. MTH1 was the only enzyme that displayed activity with N6-methyl-dATP among the enzymes tested using both 20 and 200 nm enzyme (Fig. 4). We also tested the capability of NUDT5 and NUDT14 to hydrolyze N6-methyl-dATP into N6-methyl-dADP at 20 and 200 nm enzyme concentration but no hydrolysis activity was observed (Fig. S1*A*). General activities of NUDT5, NUDT14, NUDT15, NUDT17, and NUDT18 were tested in parallel with established substrates (16–21), showing that the proteins used for these experiments were of high quality (Fig. S1, *B–E*).

### Activity of MTH1 with N6-methyl-dATP is evolutionarily conserved

**Figure 5.**
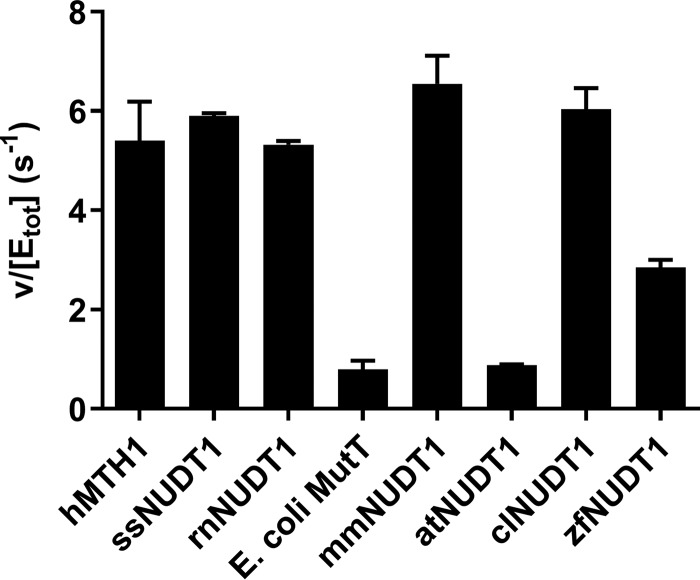
**Activity with N6-methyl-dATP is evolutionary conserved among vertebrates.** MutT homologues (MTH1 and NUDT1) from different animal species as well as *E. coli* MutT and NUDT1 from the plant *A. thaliana* were screened for hydrolysis activity with N6-methyl-dATP. Enzyme (1.25 nm) was incubated with 50 μm N6-methyl-dATP in MTH1 reaction buffer (pH 7.5) with 0.4 units/ml of PPase for 20 min at 22 °C in triplicates. P_i_ was detected using Biomol Green (Enzo Life Sciences). Absorbance at 630 nm was read after 20 min. A P_i_ standard curve was included on the plate and used to convert the assay signal to produced PP_i_. Data are shown as hydrolyzed N6-methyl-dATP (μm) divided by concentration of NUDT1 enzyme (μm) per second. The graph shows the mean ± S.D. from an experiment performed in triplicate.

To understand if the high activity of human MTH1 with N6-methyl-dATP is unique to humans or is also present in NUDT1 homologues from other species, we tested the activity of NUDT1 enzymes from humans (hMTH1), dogs (clNUDT1), pigs (ssNUDT1), rats (rnNUDT1), mice (mmNUDT1), zebrafish (zfNUDT1), the plant *Arabidopsis thaliana* (atNUDT1), and *Escherichia coli* MutT. At 50 μm N6-methyl-dATP and at a pH close to physiological (pH 7.5) all tested NUDT1 enzymes apart from *E. coli* MutT and atNUDT1 displayed notable activity toward N6-methyl-dATP (Fig. 5). This suggests that N6-methyl-dATP activity has been conserved through the evolution of vertebrates and is likely to have been present in the last common ancestor of bony fish and more developed vertebrates present more than 350 million years ago (22). To analyze the sequence similarity between the distantly related MutT homologues we performed an amino acid sequence alignment of hMTH1, *E. coli* MutT, zfMTH1, and atNUDT1 based on their crystal structures. The sequence alignment reveals a high sequence identity between hMTH1 and zfMTH1 (70.8%) despite these enzymes being from distantly related organisms (Fig. S2*A*).

### MTH1–N6-methyl-dAMP complex structure and comparison to other MTH1 structures

**Figure 6.**
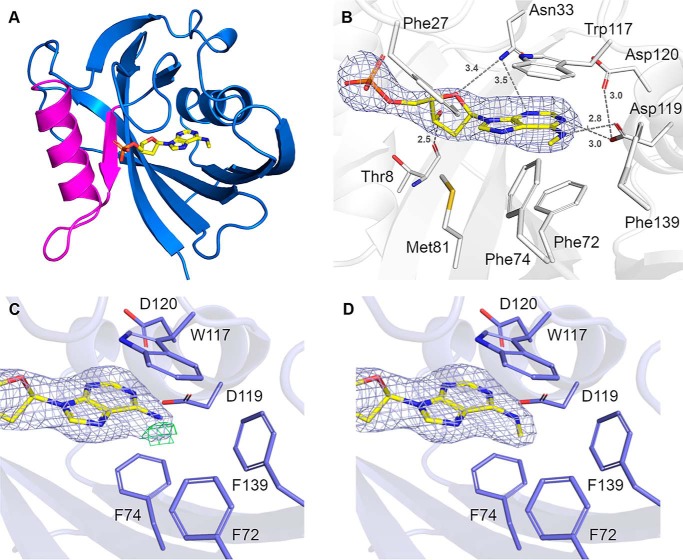
**Crystal structure of hMTH1 in complex with N6-methyl-dAMP.** *A,* overall structure of hMTH1 in ribbon representation, colored *blue*. The Nudix motif is colored *magenta*. N6-methyl-dAMP is presented as a stick model. *B,* the active site hydrogen bond network of hMTH1 with the reaction hydrolysis product N6-methyl-AMP (N6-met-AMP), with the 2*F_o_* − *F_c_* composite omit map contoured at 1.0 σ. Important binding residues and residues of the hydrophobic pocket are depicted as *sticks*; C atoms are colored *white*, O atoms *red*, N atoms *blue*, and S atoms *gold*. N6-methyl-dAMP is presented as a stick model; C atoms are colored *yellow*, O atoms *red*, N atoms *blue*, and P atoms *orange*. Hydrogen bond interactions are shown as *dashed lines* with bond distances indicated in Angstroms (Å). *C* and *D,* refinement of hMTH1 structure. The ligands (*C*) dAMP and (*D*) N6-methyl-dAMP were modeled into hMTH1 in Coot (58) following which the structures were refined using Refmac5 (59). The 2*F_o_* − *F_c_* electron density maps around the ligands following refinement are contoured at 1.0 σ (*blue*) and the *F_c_* − *F_c_* electron density maps are contoured at −2.5 σ (*red*) and +2.5 σ (*green*). Figures were produced with PyMOL (version 2.1.1, Schrödinger). Single letter amino acids are used in the figure.

**Figure 7.**
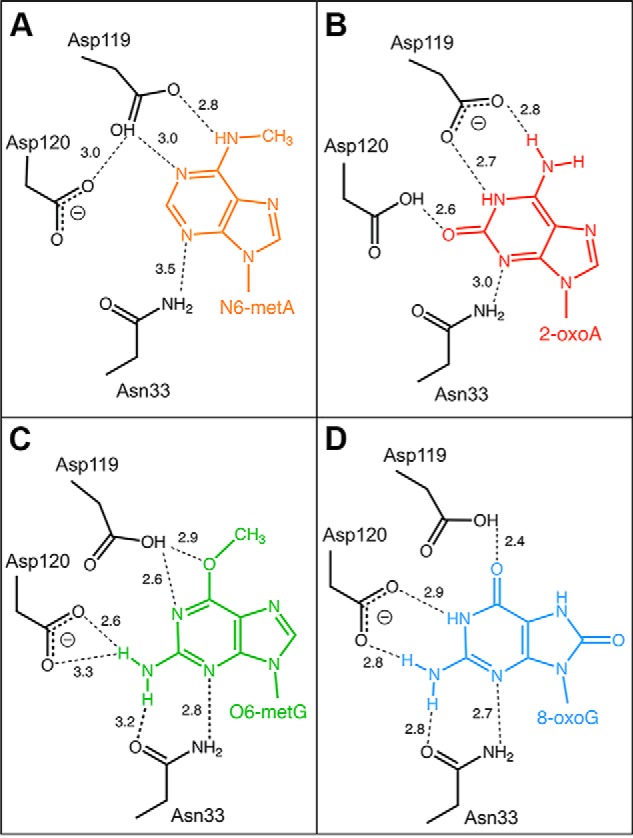
**Schematic representation of the recognition of nucleotides by hMTH1.** Hydrogen bond interactions of (*A*) N6-methyl-dAMP (N6-metA), (*B*) 2-oxo-dATP (2-oxoA) (23), (*C*) O6-methyl-dGMP (O6-metG) (10), and (*D*) 8-oxo-dGMP (8-oxoG) (24). Hydrogen bonds are shown as *dashed lines* and bond distances are given in Angstroms (Å). Deprotonated aspartates, which act as hydrogen bond acceptors, are indicated by the *minus sign*, where this is unambiguous.

To determine the binding mode of N6-methyl-dATP in the active site of MTH1, co-crystals of the nucleotide with hMTH1 were grown. The structure was solved to 2.45 Å resolution (Table S1) and the overall structure is shown in Fig. 6*A*. The structure shows clear electron density for N6-methyl-dAMP (Fig. 6*B*), indicating that hydrolysis of N6-methyl-dATP to the monophosphate form occurred during co-crystallization. The protein crystallized with four monomers in the asymmetric unit (chains A, B, C, and D). In the existing crystal form, the D-chain appears less ordered with slightly poorer electron density compared with the other monomers. The structure contains several sulfate molecules that are a result of the LiSO_4_ present in the crystallization buffer. Comparison of our structure with apo hMTH1 (PDB ID 3ZR1) resulted in low root mean square deviation values of 0.28–0.32 Å for the main chain Cα-atoms, indicating that the overall structures are virtually identical, with no structural changes induced upon ligand binding. The nucleotide-binding pocket of hMTH1 is comprised of a large number of hydrophobic amino acids including Phe-27, Phe-72, Phe-74, Met-81, Trp-117, and Phe-139 (Fig. 6*B*). The purine ring system of N6-methyl-dAMP is positioned by π-stacking interactions involving Trp-117 and Phe-72 and two hydrogen bonds with residue Asp-119, which also makes a weak interaction (3 Å) with nearby Asp-120. Asn-33 interacts weakly with the adenine base and the deoxyribose moiety of N6-methyl-dAMP as indicated by the longer bond distances of 3.4–3.5 Å. The deoxyribose group is further positioned by a hydrogen bond (2.5 Å) with the main chain carbonyl oxygen of Thr-8. There are no hydrogen bond interactions between the protein-binding pocket residues and phosphate group of the nucleotide (Fig. 6*B*). The two aspartate residues Asp-119 and Asp-120 in the hMTH1 active site have been shown to be important for MTH1 being able to bind the substrates 8-oxo-dGTP and 2-oxo-dATP (23), as well as the reaction products 8-oxo-dGMP (24) and O6-methyl-dGMP (10). In contrast to these previously solved structures, where both Asp-119 and Asp-120 interact with the modified nucleotides through hydrogen bonds, only Asp-119 makes direct hydrogen bond interactions to the nucleotide base in the MTH1 structure with N6-methyl-dAMP, with no atom of the nucleotide base of N6-methyl-dAMP within hydrogen bonding distance of Asp-120 (Fig. 7*A*). When comparing the MTH1 structure in complex with N6-methyl-dAMP to the structures with 2-oxo-dATP (PBD ID 5GHJ), 8-oxo-dGMP (PDB ID 3ZR0), and O6-methyl-dGMP (PDB ID 5OTM) these bases are shifted closer (0.53, 0.96, and 0.35 Å, respectively) to Asn-33 relative to the nucleotide base of N6-methyl-dAMP (Fig. 7, *A–D*, Fig. S3). The lack in capability to form additional hydrogen bonds with Asp-120 and Asn-33 like the other modified nucleotides (Fig. 7, *A–D*) may explain the 2.5-fold higher *K_m_* value observed for N6-methyl-dATP compared with O6-methyl-dGTP (Table 1). The hydrophobic interaction between MTH1 and the N6-methyl group of N6-methyl-dAMP pulls the base closer toward the hydrophobic subpocket, as is also observed for O6-methyl-dGMP (10). This hydrophobic interaction causes identical binding modes of O6-methyl-dGMP and N6-methyl-dAMP, despite their dramatically different hydrogen-bonding patterns (Fig. S3) indicating that this interaction makes a major contribution to the affinity for these substrates. In the structures with 8-oxo-dGTP and 2-oxo-dATP (23) the protonation state of Asp-119 and Asp-120 switches, depending on the bound substrate (Fig. 7). The protonation states of Asp-119 and Asp-120 in the N6-methyl-dAMP complex cannot be definitively stated. However, Asp-119 is likely to be protonated to interact with the N6-methyl group of the ligand as well as a nearby nitrogen from the purine ring, whereas the protonation state of Asp-120 is more ambiguous. In the interaction between Asp-119 and Asp-120 the proton could potentially be provided by either amino acid (Fig. 7).

### Substrate preference of MTH1 explained by structure analysis

The catalytic efficiency of MTH1 for hydrolyzing N6-methyl-dATP is considerably higher than for N6-methyl-ATP (Fig. 3*A* and Table 1). The additional 2-hydroxyl group of a ribose moiety would be directed toward Leu-9 and Val-83, presenting an unfavorable hydrophobic environment for this group and likely contributing to the difference in MTH1 activity between N6-methyl-dATP and N6-methyl-ATP (Fig. S4). The clear substrate preference of MTH1 for N6-methyl-dATP over dATP can be explained by the hydrophobic active site subpocket, made up by Phe-72, Phe-74, and Phe-139 (Fig. 6*B* and Fig. S5, *A* and *B*), which provides favorable hydrophobic interactions with the methyl group of N6-methyl-dATP. For dATP, which has a primary amine at the equivalent position, such a hydrophobic environment would be less favored, which may be reflected in our kinetic results that show dATP is a considerably poorer substrate for MTH1.

### N6-methyl-dATP DNA incorporation is prevented by MTH1

**Figure 8.**
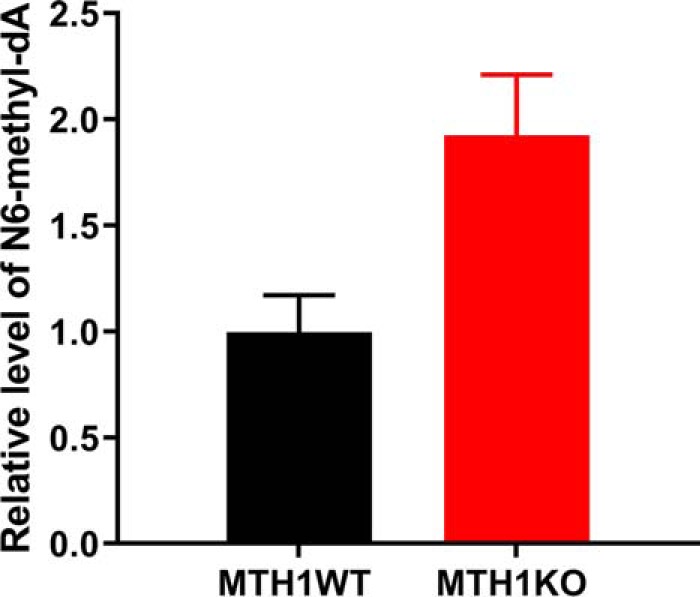
**N6-methyl-dATP is incorporated into DNA in an MTH1-dependent manner.** DNA was extracted from zebrafish MTH1KO and MTH1WT embryos developed from fertilized zebrafish eggs microinjected with N6-methyl-dATP or left untreated. DNA was analyzed for N6-methyl-dA content using LC-MS/MS. N6-methyl-dA content was normalized to N6-methyldA levels in untreated MTH1KO and MTH1WT zebrafish embryos, respectively. N6-methyl-dATP microinjected MTH1KO zebrafish embryos display a 2-fold higher N6-methyl-dA level compared with untreated embryos, whereas N6-methyl-dA DNA levels in MTH1WT zebrafish did not differ between untreated and N6-methyl-dA–microinjected embryos. This suggests that N6-methyl-dATP is incorporated into DNA and incorporation can be prevented by MTH1. The graph shows mean ± S.D., *n* = 2.

To analyze if N6-methyl-dATP is incorporated into DNA and whether this is prevented by MTH1, we used CrispR-Cas9 to delete the *mth1* gene and generate a homozygous MTH1KO zebrafish strain. Successful depletion was confirmed using sequencing. Fertilized MTH1WT and KO zebrafish eggs were microinjected with N6-methyl-dATP or left untreated. Both MTH1WT and KO zebrafish embryos tolerated the N6-methyl-dATP injection well with the same level of survival as non-injected embryos (Fig. S6*A*). We extracted DNA from untreated and microinjected WT and MTH1KO zebrafish embryos and analyzed the N6-methyl-dA content using LC-MS/MS. O6-methyl-dG levels were analyzed as a control. The N6-methyl-dA DNA levels of MTH1KO and MTH1WT microinjected with N6-methyl-dATP were normalized to the N6-methyl-dA DNA levels of untreated MTH1KO and MTH1WT zebrafish, respectively (Fig. 8). The DNA of MTH1KO zebrafish microinjected with N6-methyl-dATP contained an ∼2-fold higher relative level of N6-methyl-dA compared with the MTH1WT zebrafish microinjected with N6-methyl-dATP. This demonstrates that MTH1 prevents the incorporation of N6-methyl-dATP from the nucleotide pool into the DNA. When comparing absolute levels of N6-methyl-dA (determined as number of N6-methyl-dA per million nucleotides: MdN), N6-methyl-dATP microinjected MTH1KO zebrafish embryos displayed an ∼2-fold higher N6-methyl-dA level compared with untreated MTH1KO zebrafish embryos (Fig. S6*B*). In contrast, no difference in N6-methyl-dA DNA levels was observed between untreated and N6-methyl-dA microinjected MTH1WT zebrafish embryos (Fig. S6*C*). Interestingly, we observed a higher basal level of N6-methyl-dA in the DNA of untreated MTH1WT zebrafish embryos compared with untreated MTH1KO zebrafish embryos (Fig. S6, *B* and *C*). This observed difference may be due to a difference in the developmental stage of the zebrafish embryos caused by the lack of functional MTH1, which is reflected in different basal levels of N6-methyl-dA in the DNA at the time point of harvesting. Such changes in N6-methyl-dA levels during development have previously been observed in the *Drosophila* fly (25). Further studies of the MTH1 knockout zebrafish will be needed to definitively conclude if this occurs in zebrafish. As expected, no difference in the DNA level of O6-methyl-dG in the different treatment groups was observed (Fig. S6*D*). Altogether, this suggests that N6-methyl-dATP present in the nucleotide pool is able to be incorporated into DNA and that this can be prevented by MTH1.

## Discussion

We have identified N6-methyl-dATP as a novel substrate of MTH1 and show that MTH1 can also catalyze the hydrolysis of N6-methyl-ATP, albeit with a lower catalytic efficiency. Human MTH1 efficiently converts N6-methyl-dATP to N6-methyl-dAMP, preventing its incorporation into DNA. Comparative N6-methyl-dATP activity analyses of MTH1 homologues from a variety of vertebrate species show that the N6-methyl-dATP activity has been conserved through the evolution of vertebrate animals suggesting this activity is important to the cell.

**Figure 9.**
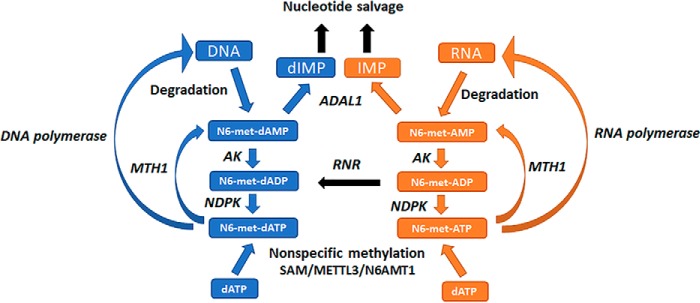
**Potential routes for cellular production and metabolism of N6-methyl-dATP and N6-methyl-ATP.** N6-methyl-dATP and N6-methyl-ATP may be produced from N6-methyl-dAMP and N6-methyl-AMP formed upon DNA and RNA degradation, respectively. This may occur through the consecutive actions of adenylate kinase (*AK*) and nucleoside diphosphate kinase or through nonspecific methylation by *S*-adenosylmethionine (*SAM*), the N6-adenosine-methyltransferase METTL3 or N6-adenine–specific DNA methyltransferase 1 (*N6AMT1*). N6-methyl-dATP and N6-methyl-ATP are hydrolyzed by MTH1 to their corresponding monophosphates and further metabolized by ADAL1 to dIMP and IMP that can then enter the nucleotide salvage pathway. Abbreviations used in the figure: *NDPK*, nucleoside diphosphate kinase; RNR, ribonucleotide reductase; METTL3, N6-adenosine methyltransferase; N6AMT1, N6-adenine-specific DNA methyltransferase 1.

For these novel MTH1 activities to be of biological significance these substrates have to be present in the cell, which raises the question of how and when N6-methyl-dATP and N6-methyl-ATP are produced in the cell. Potential routes for formation of N6-methyl-dATP and N6-methyl-ATP are depicted in Fig. 9. One source of N6-methylated adenine is the degradation of N6-methylated RNA and DNA that would give rise to N6-methyl-(d)AMP (26). N6-methyl-(d)ATP may potentially be generated through the nucleotide salvage pathway through the concerted actions of nucleotide kinases. Adenine N6-methylation plays an important role in post-transcriptional mRNA processing and is the most abundant modification found in mammalian mRNA, making up 80% of the methylations of mRNAs with an estimated frequency of 3–5 N6-methylated adenines per transcript (27). N6-methylation also occurs on rRNA, tRNA, and small nuclear RNA (27–29). Another potential route for N6-methyl-dATP and N6-methyl-ATP production is direct nonspecific methylation of dATP and ATP and other adenosine nucleotides via *S*-adenosylmethione (SAM) nonenzymatically or catalyzed by methyltransferases such as N6AMT1 and METTL3 (11, 30), which normally act on adenine in RNA and DNA (27).

Another interesting question is what relevance the N6-methyl-(d)ATP activity of MTH1 would have for the cell. N6-methylation of adenine is an established epigenetic marker in prokaryotes and has recently been reported to also have a similar function in eukaryotes (30–35). However, because the levels of N6-methyl-dA in eukaryotic DNA are low and are sometimes below the detection level of conventional LC-MS techniques (36) there is some controversy in the research field regarding the epigenetic role of N6-methyl-dA in eukaryotes. Recently, the development of SMRT sequencing of ChIP-enriched DNA has enabled the detection of N6-methyl-dA in vertebrate DNA (30, 37) and several studies suggest that N6-methyl adenine modification in DNA constitutes a second layer of epigenetic regulation, alongside cytosine methylation (38). Changes in N6-methylation levels in genomic DNA have been shown to have a major impact on diverse processes such as tumorigenesis (30, 32), development (25), cell differentiation, maintenance of cell type, and stress response (25, 33, 37, 39, 40). The association between changes in N6-methyl-dA levels and disease suggests that N6-methyl-dA is a biologically relevant DNA modification despite its evidently low abundance in eukaryotes. This implies that it is likely crucial for the cell to be able to tightly control the level of N6-methyl-dA as well as its precise locations in the genome for proper cell function (37). Indeed, N6-methylation of adenine in human DNA is regulated by the actions of the methyltransferase N6AMT1 and ALKBH1 demethylase (11, 30). Due to the low DNA levels of N6-methyl-dA even a microevent in N6-methyl-dA base modification, for example, through incorporation of N6-methyl-dATP into the DNA during replication or DNA repair, may have notable consequences for the cell by disturbing the regulation of gene expression. We show through performing *in vivo* experiments using zebrafish that N6-methyl-dATP is used as a substrate for eukaryotic DNA polymerases during replication and that it is incorporated into DNA, which confirms what previously has been shown in *in vitro* experiments (41). The significant effects of alterations in DNA levels of N6-methyladenine reported in the literature suggests that it is likely important for the cell to remove N6-methyl-dATP from the nucleotide pool and thereby prevent its incorporation into DNA.

MTH1 also displays a low but detectable activity with N6-methyl-ATP (Figs. 2 and 3 and Table 1). Even though this activity is considerably lower than for N6-methyl-dATP it cannot be excluded that it is still of some relevance, because the cellular concentration of ATP is several orders of magnitude higher than the concentration of dATP (42). Consequently, direct nonspecific methylation of ATP and dATP would produce more N6-methyl-ATP compared with N6-methyl-dATP. N6-methylation of RNA affects many processes such as transcription, splicing, translation, and stability and has been termed “the epitranscriptome” (12). N6-methylated RNA has also been shown to play roles in carcinogenesis and the determination of stem cell fate (28, 43, 44). Because N6-methyl-ATP is a substrate for eukaryotic RNA polymerases (26), MTH1 catalyzed removal of N6-methyl-ATP present in the nucleotide pool would potentially help to avoid dysregulation of this epitranscriptome by preventing N6-methyl-ATP incorporation into RNA.

The activity with N6-methyl-dATP is unique to MTH1 among human NUDIX enzymes (Fig. 4 and Fig. S1). Similarly, MTH1 was found to be the only human NUDIX enzyme that catalyzes the hydrolysis of O6-methyl-dGTP (10), suggesting that MTH1 is the sole enzyme responsible for sanitizing the nucleotide pool from methylated nucleoside triphosphates. The conversion of N6-methyl-dATP and N6-methyl-ATP to their corresponding monophosphates via MTH1 catalyzed hydrolysis would enable further conversion to inosine monophosphate and deoxyinosine monophosphate through deamination reactions catalyzed by adenosine deaminase-like protein isoform 1 (ADAL1) (Fig. 9). ADAL1 has been shown to catalyze the hydrolysis of both N6-methylated adenine and O6-methylated guanine nucleotides with similar efficiency independent of whether the sugar moiety is a ribose or a deoxyribose (45, 46). Because MTH1 also catalyzes the hydrolysis of O6-methyl-dGTP and O6-methyl-GTP (10), MTH1 and ADAL1 may act in concert to sanitize the nucleotide pool from methylated nucleotides and together convert O6-methyl-(d)GTP and N6-methyl-(d)ATP to the nontoxic nucleotides (d)GMP and (d)IMP, respectively, which can then enter the salvage pathway (Fig. 9).

We found that unlike MTH1 of animal origin, the bacterial enzyme *E. coli* MutT and the NUDT1 enzyme from the plant *A. thaliana* do not display any activity toward N6-methyl-dATP (Fig. 5). Structural analysis of the active site hydrophobic subpocket that accommodates the methyl group of N6-methyl-dATP shows that all NUDT1 homologues that catalyze the hydrolysis of N6-methyl-dATP have a phenylalanine residue in the position that corresponds to position 72 in the human enzyme (Phe-72). A sequence alignment of hMTH1, *E. coli* MutT, zfMTH1, and atNUDT1 (Fig. S2*A*) shows that while both hMTH1 and zfMTH1 have a phenylalanine in this position (Fig. S2*B*), this site is occupied by a tyrosine (Tyr-73) in *E. coli* MutT and by an asparagine (Asn-76) in atNUDT1 (Fig. S2, *C* and *D*), both being hydrophilic amino acids. Having a hydrophilic amino acid in this position presumably decreases the affinity for the methylated substrate and may at least in part explain the poor N6-methyl-dATP activity observed for *E. coli* MutT and at NUDT1. However, it may be the case that other Nudix enzymes present in *E. coli* and *A. thaliana* not included in this study have the capability to hydrolyze N6-methyl-dATP. In bacteria it would be especially advantageous to have an enzyme that sanitizes N6-methyl-dATP from the nucleotide pool, because N6-methylation of adenine is the main epigenetic mark in bacteria and is known to affect many biological processes (31, 47, 48). A potential candidate for this activity in *E. coli* is NudB, which displays activity with both canonical and oxidized dATP (1).

In summary, we have shown that N6-methyl-dATP is a novel MTH1 substrate and that N6-methyl-dATP present in the nucleotide pool is incorporated into DNA in an MTH1-dependent manner. Because N6-methyl-dA has been proposed to be an epigenetic marker, MTH1 catalyzed hydrolysis of N6-methyl-dATP may help to protect the epigenetic state of the cell. This expands the substrate collection of MTH1 and highlights the importance of MTH1 in cleansing the nucleotide pool from modified nucleotides, preventing incorporation of both oxidized and methylated nucleotides into DNA.

## Experimental procedures

### Analysis of nucleotide samples using HPLC-MS

To analyze the MTH1 catalyzed N6-methyl-dATP hydrolysis reaction in detail, N6-methyl-dATP (1 mm) was incubated with 20 nm MTH1 at 22 °C for 2.5, 5, 10, 20, 30, and 40 min in MTH1 reaction buffer (100 mm Tris acetate, pH 8.0, 40 mm NaCl, 10 mm magnesium acetate). A sample without added MTH1 was included (0 min). The reactions were stopped at the indicated time points through heat inactivation of MTH1 by incubating samples at 95 °C for 10 min followed by centrifugation at 20,000 × *g* for 10 min to remove the denatured protein by precipitation. Reaction mixture was diluted to 300 μm nucleotide with dH_2_O. Samples were analyzed using HPLC-MS utilizing an Agilent 1100 HPLC system equipped with a Hypercarb column (100 × 2.1 mm, Thermo Scientific) connected to an Agilent MSD mass spectrometer. H_2_O (containing 10 mm NH_4_HCO_3_, pH 10) and acetonitrile were used as mobile phases at a flow rate of 0.5 ml/min. The reaction mixture was separated using a gradient of 10–25% acetonitrile and a gradient time of 6.0 min. UV light absorbance in the 180–305 nm range was used for detection and masses were determined using electrospray ionization MS. To assess the stability the N6-methyl-dATP sample without added MTH1 (*T* = 0 min) was rerun after incubation at ambient temperature for 6 h.

### Production of enzymes

MTH1 was expressed in *E. coli* and purified as earlier described (24). NUDT15, NUDT17, and NUDT18 were produced as described in Ref. 21. NUDT14 and NUDT5 were generated as described in Ref. 17. NUDT1 from rat (rnNUDT1), dog (clNUDT1), pig (ssNUDT1), and mouse (mmNUDT1) were expressed and purified as reported in Ref. 49 and MTH1 from zebrafish (zfMTH1) as described in Ref. 50. *E. coli* MutT was produced as described in Ref. 6 and NUDT1 from the plant *A. thaliana* (atNUDT1) as performed in Ref. 51.

### MTH1 activity assay

Activity of MTH1 (5 or 1 nm) with 50 μm dATP (Promega), N6-methyl-dATP (Jena Bioscience), ATP (Promega), and N6-methyl-ATP (Jena Bioscience) was assayed in MTH1 reaction buffer (Tris acetate, pH 8.0, 40 mm sodium chloride, 10 mm magnesium acetate). The reaction time was 30 min and assay buffer was fortified with *E. coli* pyrophosphatase (PPase) (0.2 units/ml) to generate inorganic phosphate (P_i_) from formed PP_i_. P_i_ was detected by addition of malachite green reagent (52) followed by measurement of the absorbance at 630 nm using a Hidex plate reader. Controls without MTH1 but with PPase were included and the absorbance of control reactions was subtracted from absorbance values of assay reactions monitored in quadruplicate. A P_i_ standard curve was included on each assay plate enabling determination of the concentration of P_i_ in the wells.

### Kinetic characterization of MTH1 with N6-methyl-dATP and other substrates

Initial rates of hydrolysis of dATP, N6-methyl-dATP, N6-methyl-ATP, dGTP, and O6-methyl-dGTP into the corresponding monophosphate and PP_i_ were determined in MTH1 reaction buffer (0.1 m Tris acetate, pH 7.5, 40 mm sodium chloride, 10 mm magnesium acetate). Substrate concentrations used ranged from 0 to 200 μm for dATP and O6-methyl-dGTP, and from 0 to 300 μm for N6-methyl-dATP, N6-methyl-ATP, and dGTP. The MTH1 concentration was 1.25 nm apart from in the assay with N6-methyl-ATP activity where 10 nm MTH1 was used. Formed PP_i_ was detected using the PPiLight^TM^ Inorganic Pyrophosphate Assay (Lonza) according to the manufacturer's recommendations. A PP_i_ standard curve on the assay plate was used to calculate the amount of PP_i_ produced. Initial rates were determined in triplicate and each saturation curve experiment was performed at least three times. For determination of kinetic parameters, the Michaelis-Menten equation was fitted to the initial rate data using GraphPad Prism.

### Assessment of N6-methyl-dATP activity of human NUDIX hydrolases

Activities of 20 and 200 nm human NUDIX enzyme (MTH1, NUDT15, NUDT17, and NUDT18) were assayed with 50 μm N6-methyl-dATP or dATP in MTH1 reaction buffer (Tris acetate, pH 8.0, 40 mm sodium chloride, 10 mm magnesium acetate) by incubation with shaking at 22 °C for 20 min. 0.2 units/ml of PPase was included in the assay mixture to convert produced PP_i_ to P_i_, which was detected by addition of malachite green reagent after incubation with shaking at 22 °C followed by absorbance measurement at 630 nm after 15 min. NUDT5 and NUDT14 (20 and 200 nm) were tested for hydrolysis activity of N6-methyl-dATP (50 μm) into N6-methyl-dADP in MTH1 reaction buffer by incubation with shaking for 30 min at 22 °C. To assure high protein quality, the activity of NUDT5 and NUDT14 with their known common substrate ADP-ribose (ADPR) was measured in parallel by incubating 20 nm enzyme with 50 μm ADPR in MTH1 reaction buffer (pH 8.0) with 10 units/ml of bovine intestinal phosphatase, which cleaves off the 5′-phosphate of 5′-phosphoribose formed upon hydrolysis of ADPR. Formed P_i_ was detected using malachite green reagent as described above. To assess the general activities of NUDT15 and NUDT17, activities with 100 μm dGTP and 2-OH-dATP, respectively, using 0, 5, and 50 nm NUDT15 or 0, 25, and 250 nm NUDT17, along with 0.2 units/ml of *E. coli* PPase, were assayed. Activity of NUDT18 with 100 μm 8-oxodGDP was assayed using 0, 25, and 250 nm enzyme. Produced P_i_ was detected using malachite green reagent after incubation of NUDT15, NUDT17, and NUDT18 with respective substrate at 22 °C for 30 min in the same MTH1 reaction buffer as used for assessment of N6-methyl-dATP activity.

### Determination of N6-methyl-dATP activity among NUDT1 enzymes from different species

NUDT1 enzyme from human (hMTH1), dog (clNUDT1), pig (ssNUDT1), rat (rnNUDT1), mouse (mmNUDT1), zebrafish (zfNUDT1), *A. thaliana* (atNUDT1), and *E. coli* (*E. coli* MutT) (1.25 nm) was incubated with 50 μm N6-methyl-dATP (Jena BioScience) in MTH1 reaction buffer (0.1 m Tris acetate, pH 7.5, 40 mm sodium chloride, 10 mm magnesium acetate), and 0.4 units/ml of PPase for 20 min at 22 °C in clear 384-well-plates, in quadruplicate. P_i_ was detected using Biomol Green (Enzo Life Sciences). Absorbance at 630 nm was read after 20 min. A P_i_ standard curve was included on the plate to convert signal to P_i_ concentration.

### Crystallization and structure determination

Purified hMTH1 (10.7 mg/ml) was preincubated with 5 mm N6-methyl-dATP and 10 mm MgCl_2_. Sitting drop vapor diffusion experiments were carried out at 20 °C, where hMTH1 (stored in 20 mm HEPES, pH 7.5, 150 mm NaCl, 5% glycerol (v/v), and 0.5 mm tris(2-carboxyethyl)phosphine) was mixed in a 2:1 ratio with reservoir solution (0.1 m sodium acetate, pH 3.5, 26% PEG3350 (w/v), and 0.2 m LiSO_4_). Protein crystals were added briefly to a cryoprotectant solution consisting of the growth solution supplemented with 5 mm N6-methyl-dATP and 10% glycerol, before being flash frozen in liquid nitrogen. X-ray diffraction data were collected at station I04 of the Diamond Light Source (Oxon, UK) equipped with a PILATUS-6 M detector. A total of 360° of data were collected at 100 K at a wavelength of 1.07 Å using an oscillation angle of 0.1° and an exposure time of 0.02 s/image. Data reduction and processing were carried out using xia2 (53), DIALS (54), and Aimless (55) within the CCP4 suite (56). It was evident following data collection and processing that the crystals suffered from significant anisotropy and the resolution was therefore cut to 2.45 Å resolution. Molecular replacement was performed in Phaser (57) using the apo structure of human MTH1 (PDB ID 3ZR1) as the search model. Several rounds of manual model building and refinement using Coot (58) and Refmac5 (59) were performed, during which waters and ligands were incorporated into the structure. Data collection and refinement statistics can be found in Table S1. The structure of N6-methyl-dAMP bound hMTH1 has been deposited in the Protein Data Bank under the accession code 6QVO.

### Structure-based comparison of NUDT1 homologues to explain differences in activity

A sequence alignment of hMTH1 (UniProtKB P36639) with *E. coli* MutT (UniProtKB P08337), zfMTH1 (UniProtKB Q7ZWC3), and AtNUDT1 (UniProtKB Q9CA40) was performed using Clustal Omega through the EBI webserver (60). The resulting alignment was colored according to sequence similarity using BOXSHADE. The hMTH1 residue Phe-72 (PDB ID 6QVO), an important residue of the hydrophobic subpocket that accommodates the methyl group of N6-methyl-dAMP, was further compared with zfMTH1 (PDB ID 5OTN), AtNUDT1 (PDB ID 6FL4), and *E. coli* MutT (PDB ID 3A6T) in the program Coot (58). Pictures illustrating these superpositions were produced with PyMOL (version 2.1.1, Schrödinger).

### Production of MTH1KO zebrafish

Zebrafish lacking functional zfMTH1 (*nudt1*^uu1732^), zfMTH1KO, were created by the Genome Engineering Zebrafish facility, Scilifelab Uppsala University, using CrispR-Cas9 technology. Successful targeting of the MTH1 gene by CrispR-Cas9 was confirmed by Sanger sequencing using 5′-TCACGTCTAAGCTGC-TGACC-3′ and 5′-CCGCTTGCTCTATGGTC-TCT-3′ as sequencing primers.

### N6-methyl-dATP injection experiments and N6-methyl-dA LC-MS/MS analysis of DNA

Zebrafish were housed in standard conditions (61). To investigate if N6-methyl-dATP is incorporated into DNA by eukaryotic DNA polymerases fertilized zebrafish eggs were microinjected with N6-methyl-dATP solution as described previously (50) or left untreated. Briefly, ∼2 nl volumes of 0.3 mm N6-methyl-dATP in injection buffer (9 μm spermine, 0.21 μm spermidine, 0.3% phenol red) were injected, and eggs were distributed to plates with E3 medium (5 mm NaCl, 0.17 mm KCl, 0.33 mm CaCl_2_·2H_2_O, 0.33 mm MgSO_4_). The viability of zebrafish embryos was assessed 24 h post-injection and zebrafish were harvested after 32 h. Zebrafish DNA from the different treatment groups were isolated using the DNeasy Blood & Tissue Kit (Qiagen) according to the manufacturer's recommendations and was analyzed for N6-methyl-dA and O6-methyl-dG content, using LC-MS/MS. Prior to analysis, RNA in the DNA samples was degraded using 10 μg of RNase (Sigma-Aldrich) and incubation in 10 mm ammonium bicarbonate (pH 7.0), 1 mm magnesium chloride, and 0.1 mm deferoxamine mesylate (Santa Cruz Biotechnologies) at 37 °C for 30 min. Free nucleotides and nucleosides were removed using 30-kDa molecular mass cut-off columns (Merck) and DNA was diluted in UHPLC-grade water. Samples for LC-MS/MS were prepared by hydrolyzing up to 15 μg of DNA in 50 μl of buffer (10 mm ammonium acetate, pH 5.5, 1 mm MgCl_2_, and 0.1 mm ZnCl_2_) using 0.8 units of nuclease P1 from *Penicillium citrinum* (Sigma-Aldrich), 80 units of Benzonase® nuclease (Sigma-Aldrich), and 0.2 units of alkaline phosphatase from *E. coli* (Sigma-Aldrich) at 37 °C for 1 h. Reactions were stopped by placing the samples on ice. Proteins were precipitated by adding three volumes of ice-cold acetonitrile followed by centrifugation at 16,000 × *g* for 30 min. Supernatants were then lyophilized at −80 °C. Finally, the samples were re-dissolved in 30 μl of water, of which 5 μl was diluted 5000-fold and used for measuring the four canonical nucleosides and 20 μl was used to measure modified nucleosides using LC-MS/MS analysis. Modified nucleosides were analyzed using an Agilent 6495 triple quadrupole LC/MS/MS system with an Agilent EclipsePlusC18 RRHD column (2.1 × 150 mm, 1.8-μm particle size). The mobile phases used were (A) UHPLC-grade water and (B) UHPLC-grade methanol, both containing 0.1% UHPLC-grade formic acid. The HPLC method used a flow rate of 300 μl/min with 5% B for 2.5 min, followed by a gradient to 13% B at 3 min, a ramp to 17.16% B at 5.5 min, hold at 35% B from 5.5 to 7 min, a ramp to 5% at 8 min, and finally equilibration with 5% B from 7 to 11.5 min. Unmodified nucleosides were measured on an API5500 triple quadrupole mass spectrometer (Applied Biosystems) with an Acentis® Express C18 column (0.5 × 150 mm, 2.7-μm particle size). The HPLC method used a flow rate of 150 μl/min with an isocratic flow of 25% B for 3 min with the column heated to 40 °C. The mass transitions used were 266.1 → 150, 282.1 → 166.1, 252.1 → 136, 228.1 → 111.9, 268.1 → 152, and 243.1 → 127 *m*/*z* for N6-met-dA, O6-met-dG, dA, dC, dG, and dT, respectively.

### Data availability

The protein structure presented in this paper has been deposited in the Protein Data Bank (PDB) under the accession code 6QVO. All remaining data are contained within the article.

## Author contributions

E. R. S. and A.-S. J. formal analysis; E. R. S., K. S. V., L. B., A. S., P. S., and A.-S. J. investigation; E. R. S., K. S. V., L. B., A. S., and A.-S. J. methodology; E. R. S., K. S. V., L. B., A. S., and A.-S. J. writing-original draft; E. R. S., U. W. B., T. H., P. S., and A.-S. J. writing-review and editing; U. W. B., T. H., and P. S. supervision; U. W. B. project administration; T. H. and P. S. funding acquisition; A.-S. J. wrote the manuscript with support from E. R. S.; A. S. J. conceived and designed the study as well as performed and analyzed biochemical experiments.

## Supplementary Material

Supporting Information

## Footnotes

3 The abbreviations used are:

MTH1: MutT homologue 1
ADAL1: adenosine deaminase-like protein isoform 1
MTH1KO: MTH1 knockout
MTH1WT: MTH1 wildtype
PPase: pyrophosphatase
UHPLC: ultra performance HPLC
PDB: Protein Data Bank.
