# Supplementary material for: MutT homologue 1 (MTH1) removes N6-methyl-dATP from the dNTP pool
Source: J Biol Chem. 2020 Mar 6;295(15):4761–72. doi: 10.1074/jbc.RA120.012636 (PMC7152754; doi:10.1074/jbc.RA120.012636)
Supplement: Supporting Information [file supp_295_15_4761__index.html]

MutT homologue 1 (MTH1) removes N6-methyl-dATP from the dNTP pool — MTH1 catalyzes N6-methyl-dATP hydrolysis — MutT homologue 1 (MTH1) removes N6-methyl-dATP from the dNTP pool — EDITORS' PICK: MTH1 catalyzes N6-methyl-dATP hydrolysis — Supporting Information 

# MutT homologue 1 (MTH1) removes N6-methyl-dATP from the dNTP pool

## Supporting Information

- Supporting Information (to be published online) to MutT homologue 1 (MTH1 removes N6-methyl-dATP from the dNTP pool - Supporting information containing supplementary table and supplementary figure 1-6
