# Supplementary material for: MutT homologue 1 (MTH1) removes N6-methyl-dATP from the dNTP pool
Source: J Biol Chem. 2020 Mar 6;295(15):4761–72. doi: 10.1074/jbc.RA120.012636 (PMC7152754; doi:10.1074/jbc.RA120.012636)
Supplement: Supporting Information [file supp_RA120.012636_158181_2_supp_485693_q6lz0c.pdf]

Supporting information

Supplementary Table 1. Data collection and refinement statistics

**Data collection**	
PDB code	
Space group	$P2_1$
Cell dimensions	
a, b, c (Å)	35.5, 67.1, 117.7
α, β, γ (°)	90.0, 94.7, 90.0
No. of observations	139304 (16002)
No. of unique reflections	20436 (2307)
Resolution (Å)	58.6−2.45 (2.55−2.45)
R_{merge} (%)	9.6 (34.2)
CC(1/2) (%)	99.8 (97.4)
$<I / \sigma(I)>$	9.2 (3.2)
Completeness (%)	100 (100)
Redundancy	6.8 (6.9)
**Refinement**	
Resolution (Å)	58.6−2.45
No. of reflections	19444
R_{work}/R_{free}	23.7/28.5
No. of atoms:	
Protein	4935
N^6-Methyl-dAMP	92
Other ligands	36
Water	88
B-factors:	
Protein	58.0
N^6-Methyl-dAMP	72.7
Other ligands	89.1
Water	44.4
R.m.s. deviations:	
Bond lengths (Å)	0.007
Bond angles (°)	1.21
Ramachandran plot (*N*, %)	
Favoured	100
Allowed	2

Values in parenthesis are for the highest resolution shell.

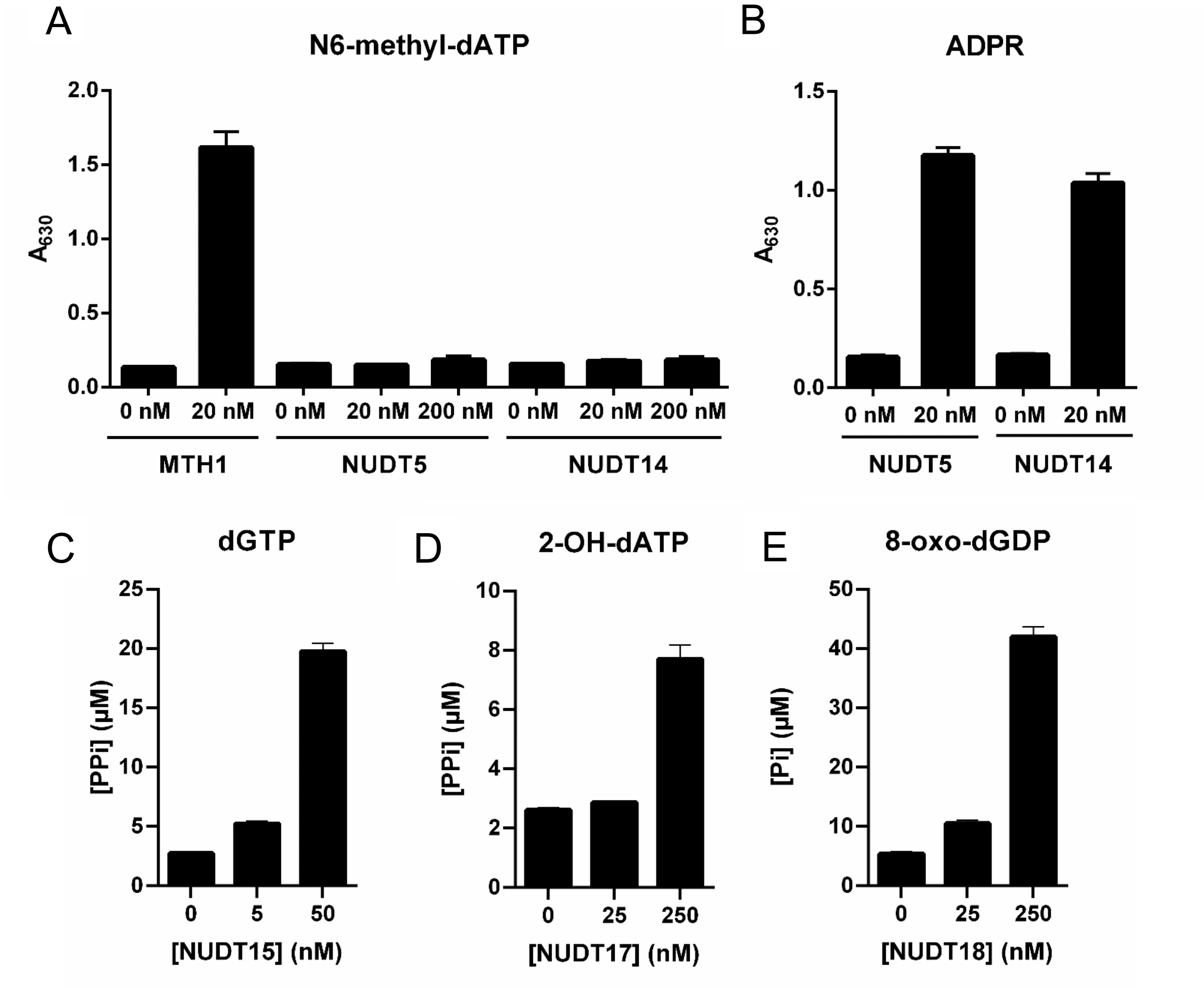

Supplementary Figure 1. Activity with N6-methyl-dATP is unique to MTH1 among closely related human NUDIX enzymes. (A) Hydrolysis activity of NUDT5 and NUDT14 with N6-methyl-dATP to N6-methyl-dADP was assayed using 0, 20 and 200 nM enzyme. Activity of NUDT5 and NUDT14 with N6-methyl-dATP at 200 nM was slightly above background. Control reactions with validated substrates were performed and enzymes were found to be active. B) NUDT5 and NUDT14 (20 nM) were assayed with 50 µM ADP-ribose (ADPR). (C) NUDT15 was assayed with 100 µM dGTP at 5 and 50 nM enzyme. (D) Activities of NUDT17 and (E) NUDT18 preparations were assessed using 100 µM 2-OH-dATP and 100 µM 8-oxo-dGDP, respectively at 0, 25 and 250 nM enzyme. D) Activities were assayed in MTH1 reaction buffer, pH 8.0. Data are shown as average and SD of assay samples performed in at least triplicate.

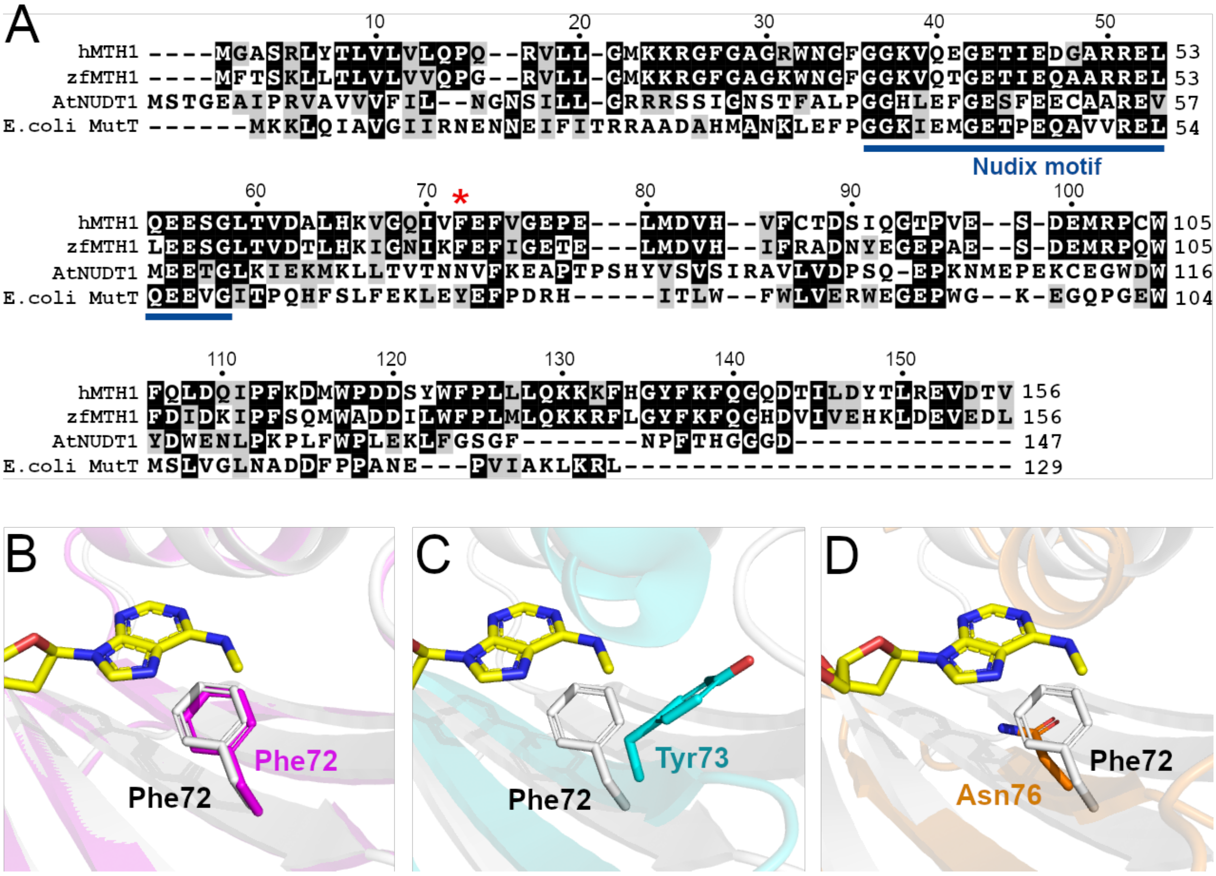

Supplementary Figure 2. (A) Sequence alignment of hMTH1 (UniProtKB: P36639), MutT (UniProtKB: P08337), zfMTH1 (UniProtKB: Q7ZWC3) and AtNUDT1 (UniProtKB: Q9CA40). Identical residues are shaded black, while grey shading indicated residues with conserved physicochemical properties. The highly conserved Nudix motif (GX_5EX_7REUXEEXGU, where U is a hydrophobic residue) is shown. The hMTH1 residue Phe72, an important residue of the hydrophobic sub-pocket, which accommodates the methyl group of N6-methyl-dATP is indicated by red asterisk. Structural superpositions comparing the Phe72 position hMTH1-N6-methyl-dAMP (white) with **(B)** zfMTH1 (pink) **(C)** MutT (cyan) and **(D)** AtNUDT1 (orange). Throughout panels B-D, N6-methyl-dAMP is shown as a stick model; C atoms are colored grey, O atoms red, N atoms blue and P atoms orange. The structural superpositions shown in panels B-D were illustrated using the program PyMOL.

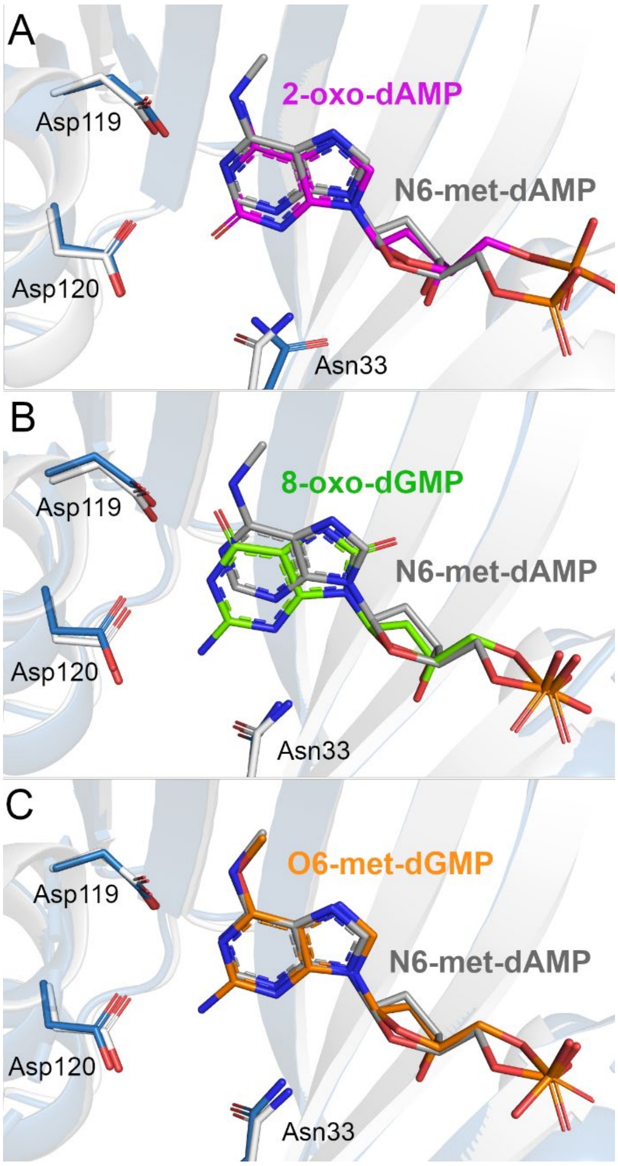

Supplementary Figure 3. Comparison of N6-methyl-dAMP (N6-met-dAMP) bound hMTH1 with existing nucleotide bound structures. Throughout panels A-C, N6-met-dAMP bound hMTH1 is presented as a cartoon (white). The amino acids D119, D120 and N33 which are key to ligand recognition are shown as sticks; C atoms are colored white, O atoms red and N atoms blue. hMTH1 bound with other modified nucleotides is shown as a blue cartoon. D119, D120 and N33 have the same color scheme as for N6-met-dAMP bound hMTH1, except that C atoms are colored blue. N6-met-dAMP is shown as a stick model; C atoms are colored grey, O atoms red, N atoms blue and P atoms orange. **(A)** Superposition of N6-met-dAMP and 2-oxo-dAMP bound to hMTH1 (PDB ID: 5GHJ). 2-oxo-dAMP is shown as a stick model; C atoms are colored magenta. **(B)** Superposition of N6-met-dAMP and 8-oxo-dGMP bound to hMTH1 (PDB ID: 3ZR0). 8-oxo-dGMP is shown as a stick model; C atoms are colored green. **(C)** Superposition of N6-met-dAMP and O6-methyl-dGMP (O6-met-dGMP) bound to hMTH1 (PDB ID: 5OTM). O6-met-dGMP is shown as a stick model; C atoms are colored orange and P atoms light orange.

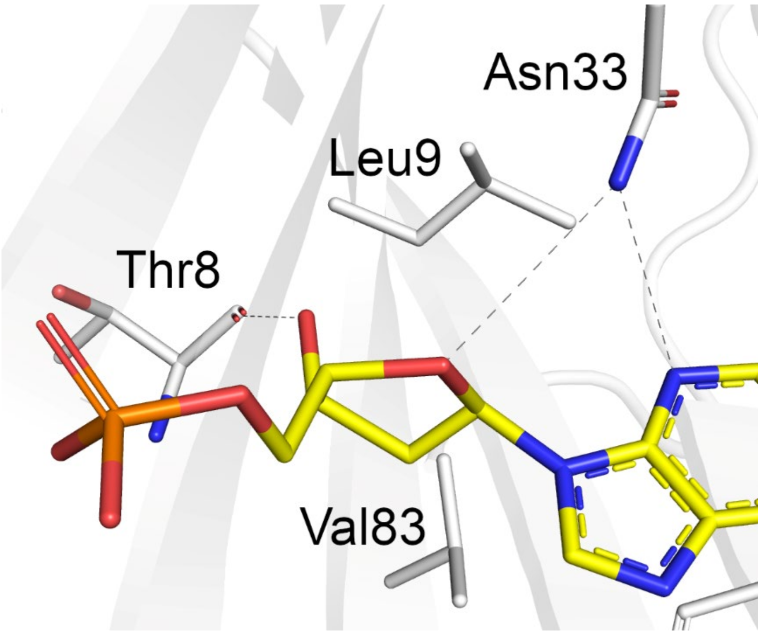

Supplementary Figure 4. Crystal structure of hMTH1 in complex with N6-methyl-dAMP highlighting the deoxyribose moiety. Important residues are depicted as sticks; C atoms are colored white, O atoms red and N atoms blue. N6-met-dAMP is presented as a stick model; C atoms are colored yellow, P atoms are orange. Hydrogen bond interactions are shown as dashed lines. Figures were produced with PyMOL (v.2.1.1, Schrödinger).

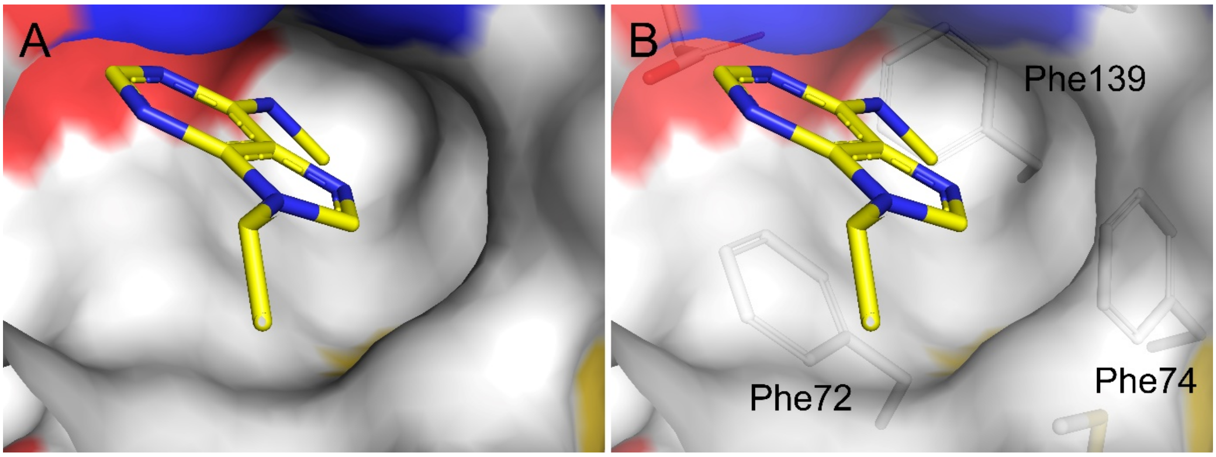

Supplementary Figure 5. Active site hydrophobic pocket of MTH1. **(A)** Surface representation of the hydrophobic pocket which accommodates the N6-methyl group of N6-methyl-dAMP. The surface is colored according to atom type; carbon atoms white, nitrogen atoms blue, oxygen atoms red and sulfur atoms yellow. N6-methyl-adenine is shown as a stick model with carbon atoms colored yellow and nitrogen atoms colored blue. **(B)** The same as Panel A but with the surface displayed with 20 % transparency. Phenylalanines which comprise the hydrophobic pocket are labelled. Figures were produced in PyMOL (v.2.1.1, Schrödinger).

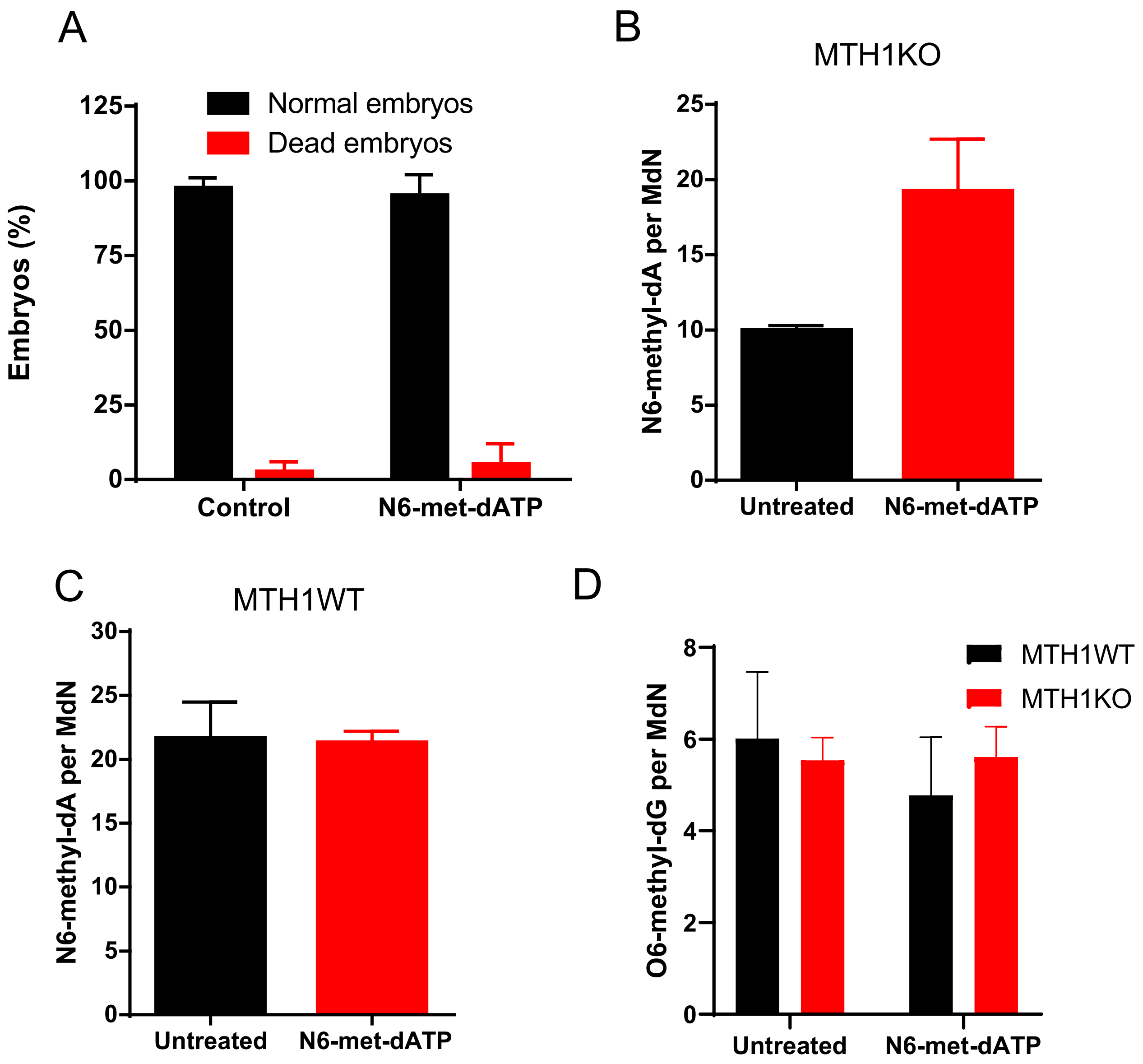

Supplementary Figure 6. **(A)** Injection of N6-methyl-dATP does not affect survival of zebrafish embryos. Fertilized MTH1WT zebrafish eggs were injected with N6-methyl-dATP and zebrafish embryos were scored for survival after 24 h. Levels of N6-methyl-dA in untreated and N6-methyl-dATP microinjected zebrafish DNA in **(B)** MTH1KO and **(C)** MTH1WT zebrafish embryos measured per million nucleotides (MdN). **(D)** Levels of O6-methyl-dG of MTH1WT and MTH1KO zebrafish in DNA isolated from untreated and N6-methyl-dATP microinjected embryos measured per MdN showing that levels of O6-methyl-dG differ neither between zebrafish injected with N6-methyl-dATP or untreated nor between MTH1WT and MTH1KO zebrafish embryos. Graphs show means with SD, n=2.
